# *RUNX1* Mutations in Inherited and Sporadic Leukemia

**DOI:** 10.3389/fcell.2017.00111

**Published:** 2017-12-20

**Authors:** Dana C. Bellissimo, Nancy A. Speck

**Affiliations:** Department of Cell and Developmental Biology, Perelman School of Medicine, Abramson Family Cancer Research Institute, Institute for Regenerative Medicine, University of Pennsylvania, Philadelphia, PA, United States

**Keywords:** *RUNX1*, leukemia, myeloid neoplasms, leukemia predisposition, familial platelet disorder with predisposition for acute myeloid leukemia, pre-leukemia

## Abstract

*RUNX1* is a recurrently mutated gene in sporadic myelodysplastic syndrome and leukemia. Inherited mutations in *RUNX1* cause familial platelet disorder with predisposition to acute myeloid leukemia (FPD/AML). In sporadic AML, mutations in *RUNX1* are usually secondary events, whereas in FPD/AML they are initiating events. Here we will describe mutations in *RUNX1* in sporadic AML and in FPD/AML, discuss the mechanisms by which inherited mutations in *RUNX1* could elevate the risk of AML in FPD/AML individuals, and speculate on why mutations in *RUNX1* are rarely, if ever, the first event in sporadic AML.

## Introduction

Acute leukemia is caused by the acquisition of mutations in hematopoietic stem and/or progenitor cells (HSPCs) that promote their clonal expansion and impair downstream differentiation. The earliest initiating step in leukemia is a mutation that generates a hematopoietic stem cell (HSC) that is, in some poorly understood way, primed for leukemic transformation by secondary mutations. The mutations in HSCs that initiate sporadic leukemia in adults generally occur in genes encoding epigenetic regulatory proteins such as *DNMT3A, ASXL1, IDH2*, and *TET2* (Papaemmanuil et al., 2016). Disease progression is caused by the acquisition of secondary mutations in HSCs containing initiating mutations. These secondary, driver mutations involve genes encoding several functional categories of proteins including transcription factors (e.g., *CEBPA, RUNX1, GATA2*, and *ETV6*), signaling molecules (*FLT3, NRAS, PTPN11, KRAS, KIT, CBL*, and *NF1*), splicing factors (*SRSF2, SF3B1*, and *U2AF1*), and proteins with other functions (*NPM1, SMC1A*) (Cancer Genome Atlas Research Network et al., 2013; Papaemmanuil et al., 2016).

The order of mutations in sporadic acute leukemia in adults, where mutations in epigenetic regulators generally precede those in genes encoding other categories of proteins, is upended in individuals who have inherited a leukemia predisposition gene. Leukemia predisposition genes are constitutional mutations that greatly elevate the lifetime risk of leukemia, which in the general population is 1.5% (Howlader et al., 1975–2014), but in individuals with inherited leukemia disposition genes, the risk of myeloid malignancy is much higher depending on the mutation and other genetic, epigenetic, and environmental factors (Owen C. et al., 2008; Liew and Owen, 2011; West et al., 2014). For instance, the lifetime risk of myeloid malignancy is about 44% with germ line *RUNX1* mutations; however, the lifetime risk approaches nearly 100% for germ line *CEBPA* mutations (Godley, 2014). The importance of these inherited mutations in leukemia, their distinct clinical features, and the implications for treatment were recently recognized by the World Health Organization in their 2016 revision to the classification scheme for myeloid neoplasms and acute leukemia, which includes a new category of “myeloid neoplasms with germ line predisposition” (Arber et al., 2016; Table 1). Of note, mutations in several of the genes that confer leukemia predisposition (e.g., *CEBPA, ETV6, GATA2, NF1, RUNX1, PTPN11, CBL*, and *RAS*) are also frequently found in sporadic acute myeloid leukemia (AML) or myelodysplastic syndrome (MDS) (Cancer Genome Atlas Research Network et al., 2013). However, an important distinction, for which the mechanistic basis is not well understood, is that these inherited mutations are rarely initiating events in sporadic AML in adults but are, by definition, the initiating events in myeloid neoplasms with germ line predisposition.

**Table 1:** World Health Organization classification of myeloid neoplasms with germ line predisposition.

**Myeloid neoplasm classification**	**Genes involved**	**Recurrently mutated in sporadic AML, largely initiating or secondary event**
Myeloid neoplasms without a preexisting disorder or organ dysfunction	*CEBPA* *DDX41*	Yes, secondary NoNo
Myeloid neoplasms and preexisting platelet disorders	*RUNX1* *ANKRD26* *ETV6*	Yes, secondary No NoYes, secondary
Myeloid neoplasms and other organ dysfunction		
Germ line *GATA2* mutation	*GATA2*	Yes, secondary
BM failure syndromes^*^	*Multiple*	No
Telomere biology disorders	*TERT, TERK*	No
JMML associated with neurofibromatosis, Noonan syndrome or Noonan syndrome- like disorders, Down syndrome	*NF1*, *PTPN11*, *CBL, KRAS*	Yes, secondary

* *Includes Fanconi anemia (FANCA, FANCB, FANCC, FANCD1, FANCD2, FANCE, FANKF, FANCG, FANCI, FANCJ, FANCL, FANCM, FANCN), dyskeratosis congenita (DKC1, TERC, TERT, TIN2, NOP10, NHP2), Schwachman-Diamond syndrome (SBDS), Diamond Blackfan anemia (RPS19, RPS24, RPS17, RPL5, RPL11, RPL35A, RPS7, RPS10, RPS26, GATA1), congenital amegakaryocytic thrombocytopenia (MPL), and severe congenital neutropenia (ELA2, GFI1, HAX1*).

Here we will focus on *RUNX1*, a leukemia predisposition gene that is also frequently mutated in sporadic leukemia. We will describe the inherited and acquired mutations in *RUNX1* and speculate on why mutations in *RUNX1* can be initiating events when inherited but are rarely initiating events in sporadic leukemia.

## Somatic mutations in *RUNX1*

*RUNX1* encodes a sequence-specific transcription factor that is essential for HSC formation in the conceptus and is important for the differentiation of cells of the lymphoid, myeloid, and megakaryocytic lineages (Cai et al., 2000; Ichikawa et al., 2004; Growney et al., 2005; Tober et al., 2016). *RUNX1* is a recurrent target of somatic mutations in *de novo* AML, myelodysplastic syndrome (MDS), acute lymphocytic leukemia (ALL), atypical chronic myeloid leukemia (aCML), and secondary AML (Mangan and Speck, 2011). There are two broad categories of mutations in *RUNX1*: monoallelic chromosomal translocations and mono- or biallelic somatic mutations. The most common chromosomal translocations are t(8;21)(q22;q22) in *de novo* AML and t(12;21)(p13;q22) in acute lymphocytic leukemia (B-ALL). Both of these translocations are initiating events in sporadic leukemia and can be acquired *in utero* as evidenced by their presence in Guthrie card samples from newborns diagnosed in childhood with AML or B-ALL (Wiemels et al., 1999, 2002). The t(8;21) and t(12;21) translocations generate fusion proteins (RUNX1-RUNX1T1 and ETV6-RUNX1, respectively) with neomorphic activity (Miyoshi et al., 1991, 1993; Chang et al., 1993; Nucifora et al., 1993; Golub et al., 1995; Romana et al., 1995; Shurtleff et al., 1995; Yergeau et al., 1997; Okuda et al., 1998; Wildonger and Mann, 2005; Schindler et al., 2009). Both translocations confer a favorable prognosis in their respective diseases (Grimwade et al., 1998; Byrd et al., 2002; Schlenk et al., 2003). AML with t(8;21)(q22;q22) or with inv(16)(p13;q22) or t(16)(p13;q22), which disrupt *CBFB* the non-DNA-binding partner of RUNX1, are included under the category of “AML with recurrent genetic abnormalities” in the 2016 WHO classification scheme (Arber et al., 2016) and together are often referred to as “core-binding factor acute myeloid leukemia” (CBF-AML).

Mono- and biallelic mutations in *RUNX1* include deletions, missense, splicing, frameshift, and nonsense mutations. These mutations are mechanistically distinct from the chromosomal translocations and confer a worse prognosis (Osato et al., 1999; Imai et al., 2000; Harada et al., 2003; Steensma et al., 2005; Gelsi-Boyer et al., 2008; Kuo et al., 2009; Bejar et al., 2011; Mangan and Speck, 2011; Gaidzik et al., 2016). Some mutations truncate the RUNX1 protein N-terminal to or within the DNA-binding domain and consequently inactivate the protein. Other mutations confer weak dominant negative activity to RUNX1. For example, mutations in DNA-contacting residues that disrupt DNA binding without perturbing the structure of the DNA-binding domain behave as weakly dominant negative mutations (Michaud et al., 2002; Matheny et al., 2007; Owen C. J. et al., 2008; Preudhomme et al., 2009; Bluteau et al., 2011). The mechanism by which this occurs is not known but probably involves RUNX1 binding to and sequestering a limiting protein through an interaction that requires RUNX1 to be properly folded. A possible candidate for this limiting protein is the non-DNA-binding subunit partner of the RUNX proteins, CBFβ, which associates with the RUNX1 DNA-binding domain. Another type of dominant negative mutation removes the C-terminal transactivation domain while leaving the DNA-binding domain intact, which allows mutant RUNX1 to occupy its target sites and block occupancy and transactivation by full length RUNX proteins (RUNX1, RUNX2, and RUNX3; Harada et al., 2003; Satoh et al., 2008). For simplicity's sake, we will refer to both the weakly dominant negative and inactivating *RUNX1* mutations as “mutations” to distinguish them from the chromosomal translocations. AML with mono- and biallelic *RUNX1* mutations has been provisionally classified by the WHO as “AML with mutated *RUNX1*” (distinct from CBF-AML) to reflect the possible worse prognosis as compared to other AML types (Tang et al., 2009; Gaidzik et al., 2011, 2016; Schnittger et al., 2011; Mendler et al., 2012; Arber et al., 2016).

The order of mutation acquisition in sporadic AML can be discerned by whole-genome sequencing, which reveals not only which mutations are present but also the allelic fraction of each mutation. Clonal mutations, present in all leukemia cells, represent initiating events, while subclonal mutations are secondary events. Whereas mutations in epigenetic modifiers such as *DNMT3A, ASXL1, IDH2*, and *TET2* have been consistently identified as early mutations in studies examining the temporal order of mutation acquisition in sporadic AML (Jan et al., 2012; Krönke et al., 2013; Corces-Zimmerman et al., 2014; Shlush et al., 2014; Hirsch et al., 2016; Papaemmanuil et al., 2016), *RUNX1* mutations have rarely been identified as “early” or “initiating” mutations in these studies (Jan et al., 2012; Corces-Zimmerman et al., 2014; Shlush et al., 2014; Hirsch et al., 2016; Papaemmanuil et al., 2016). The largest and most recent analysis of the order of mutation acquisition was conducted using 1540 *de novo* AML samples and validated the previous identification of *DNMT3A, ASXL1, IDH2*, and *TET2* as the earliest mutations in sporadic AML (Papaemmanuil et al., 2016). In this large analysis, as in prior smaller analyses, acquired *RUNX1* mutations were not identified as the earliest mutations and were rarely clonal. In a calculation to determine the relative order of mutation acquisition based on pairwise precedences, sporadic *RUNX1* mutations occurred 11th out of 28 genes analyzed. Thus, *RUNX1* mutations in sporadic AML are usually intermediate secondary events that drive disease progression (Jan et al., 2012; Corces-Zimmerman et al., 2014; Shlush et al., 2014; Hirsch et al., 2016; Papaemmanuil et al., 2016).

Myelodysplastic syndrome (MDS) is a clonal hematopoietic disorder characterized by ineffective hematopoiesis and is a harbinger of AML in approximately 25–30% of patients (Tefferi and Vardiman, 2009; Steensma, 2015). MDS is characterized by cytopenias of one or more peripheral blood lineages, bone marrow cells with dysplastic features, and ≤19% blast cells (Arber et al., 2016). Mutations in *RUNX1* are usually secondary events in MDS, although in a small number of patients they were identified as initiating events in clonal analyses (Papaemmanuil et al., 2013; Thota et al., 2014; da Silva-Coelho et al., 2017).

The paucity of initiating *RUNX1* mutations is particularly striking in the age-related phenomenon of clonal hematopoiesis of indeterminant potential (CHIP) (Genovese et al., 2014; Jaiswal et al., 2014; Xie et al., 2014). CHIP is caused by a mutation in an HSC that causes it to selectively expand in the bone marrow relative to normal HSCs. The presence of CHIP is detected by an increase in the fraction of a mutated allele in peripheral blood (Busque et al., 2012; Genovese et al., 2014; Jaiswal et al., 2014; Xie et al., 2014; McKerrell et al., 2015). Despite having an expanded HSC clone, individuals with CHIP have normal hematopoiesis. The prevalence of CHIP increases with age—it is rare in individuals less than 40 years of age, increases to approximately 5–10% incidence in people over the age of 70, and reaches an incidence of 20% in individuals ≥90 years of age (Genovese et al., 2014; Jaiswal et al., 2014; McKerrell et al., 2015). CHIP confers a modestly elevated risk of MDS and AML (0.5–1% per year). The most common mutations in CHIP are the same as those identified as initiating mutations in sporadic adult AML: *DNMT3A, TET2*, and *ASXL1* (Genovese et al., 2014; Jaiswal et al., 2014; Xie et al., 2014). CHIP is thought to represent a first step in leukemogenesis in adults that at a low, but discernably elevated, rate progresses to leukemia. Strikingly, in three separate studies not a single mutation was found in *RUNX1* in individuals with CHIP (Genovese et al., 2014; Jaiswal et al., 2014; Xie et al., 2014). Mutations in several other germ line leukemia predisposition genes (*CEBPA, GATA2, ETV6*) were also rare or undetected.

*RUNX1* mutations are more common in clonal cytopenia of undetermined significance (CCUS) (Kwok et al., 2015; Malcovati et al., 2017). CCUS is a subcategory of idiopathic cytopenias of undetermined significance (ICUS) (Valent et al., 2012; Cargo et al., 2015; Kwok et al., 2015), which include unexplained cytopenias that fail to meet the diagnostic criteria for MDS. CCUS is distinct from CHIP by virtue of its abnormal hematopoiesis. Approximately 2–4% of CCUS patients had *RUNX1* mutations (Kwok et al., 2015; Malcovati et al., 2017).

In summary, mutations in *RUNX1* are rarely initiating events in AML, have not been observed in CHIP, and have been identified at a low frequency in MDS and CCUS.

## Inherited mutations in *RUNX1*

Inherited mono-allelic *RUNX1* mutations are associated with “familial platelet disorder with predisposition to AML” (FPD/AML) (Song et al., 1999). FPD/AML individuals usually present with mild to moderate thrombocytopenia and bleeding disorders (epistaxis, easy bruising, excessive bleeding during minor surgery, menorrhagia) (Luddy et al., 1978). The bleeding disorder is caused by impaired proplatelet formation, functional aspirin-like platelet activation defects, and abnormal megakaryocyte differentiation and polyploidization. FPD/AML individuals also have a strikingly elevated (~44% lifetime risk) of MDS or acute leukemia (AML and to a lesser extent T-ALL) (Godley, 2014). Since the bleeding disorder is usually manageable and in most individuals does not greatly compromise the quality of life, bone marrow transplantation from a non-affected family member or an unrelated matched donor is generally not recommended unless the patient has leukemia (University of Chicago Hematopoietic Malignancies Cancer Risk Team, 2016).

The ability to monitor HSC expansion by changes in variant allele frequencies in FPD/AML individuals by next generation sequencing may offer the opportunity to intervene at the pre-leukemic stage, prior to the appearance of overt MDS or leukemia. Knowing when to intervene and treat a healthy individual at an early stage of disease, however, requires knowing which secondary mutations are likely to lead to disease progression. In sporadic leukemia, it is assumed that the most potent cooperating mutations are those that most frequently co-occur due to selection. For *RUNX1* in sporadic leukemia, these include mutations in *ASXL1, BCOR, KMD2A, PHF6*, and *SRFS2*, and less significantly in *IDH2, STAG2, SF3B1*, and trisomy 13 (Gaidzik et al., 2016; Papaemmanuil et al., 2016). On the other hand, *RUNX1* mutations are inversely correlated with mutations in *CEBPA, NPM1, TP53*, t(8;21)(q22;q22), inv(16)(p13;q22)/t(16)(p13;q22), and t(15;17)(q24;q21), presumably because the mutations are either functionally redundant or antagonistic. Other common MDS or AML mutations (e.g., in *DNMT3A*) were neither enriched nor inversely correlated with *RUNX1* mutations.

Unfortunately, the rarity of FPD/AML makes definitive large-scale sequencing studies impossible, and the handful of smaller studies that have been conducted reported somewhat different results. One group sequenced AML samples from 9 Japanese FPD/AML patients and reported somatic mutations in *CDC25C* in 4 patients, concluding that somatic mutations in *CDC25C* (which are rarely observed in sporadic AML) were the most common genetic event in AML arising from FPD/AML (Yoshimi et al., 2014; Sakurai et al., 2016). A second group analyzed somatic mutations in 8 leukemia patients from four French FPD/AML families, identified loss of function mutations in the second *RUNX1* allele in 6 patients, and concluded that mutation of the remaining wild type *RUNX1* allele was the most common secondary event (Antony-Debré et al., 2016). Strikingly, the French group found no mutations in *CDC25C*. A third group (Churpek et al.) from the United States analyzed somatic mutations in 7 FPD/AML patients with MDS or AML and found no mutations in either *CDC25C* or in the second *RUNX1* allele, but instead, identified mutations in a collection of other genes including *BCOR, PHF6, DNMT3A, TET2, CREBBP, U2AF1, NUP214, SMC3*, and *PDS5B* (Churpek et al., 2015).

In the Churpek et al. study, the peripheral blood of 9 FPD/AML individuals with no evidence of leukemia was also analyzed (Churpek et al., 2015). Six of these 9 individuals had clonal hematopoiesis, and a missense mutation in *DNMT3A* was found in one person (Churpek et al., 2015). A *DNMT3A* mutation was also identified in an asymptomatic FPD/AML individual in a separate study (Antony-Debré et al., 2016). The asymptomatic FPD/AML individuals with evidence of clonal hematopoiesis ranged in age from 8 to 54 (Churpek et al., 2015), whereas CHIP in the general population is extremely rare in this age group (Genovese et al., 2014; Jaiswal et al., 2014; Xie et al., 2014). It appears that the acquisition of secondary mutations is greatly accelerated in FPD/AML individuals and presumably contributes to the elevated risk of leukemia in these patients. An important question raised by these studies is why clonal hematopoiesis is so greatly accelerated in individuals with FPD/AML. In the next several sections we will describe experimental data that begin to address this question.

## Defects in DNA repair pathways in *RUNX1* mutant cells

Inefficient DNA repair could contribute to the rapid accumulation of mutations seen in non-leukemic FPD/AML patients. This could result from the dysregulated expression or activity of proteins in DNA repair pathways, which include mismatch repair (MMR), base excision (BER), nucleotide excision (NER), double-strand break repair [homologous recombination (HR), non-homologous end joining (NHEJ), alternative non-homologous end-joining (Alt-NHEJ)], and interstrand DNA crosslink repair by the Fanconi Anemia (FA) / BReast CAncer susceptibility (BRCA) pathway (Cheung and Taniguchi, 2017). Thus far, RUNX1 has been implicated in regulating the BER, HR, and FA/BRCA pathways.

Most evidence for RUNX1's role in DNA repair has been generated using cells expressing the neomorphic RUNX1-RUNX1T1 (AML1-ETO) protein. Several studies showed that expression of RUNX1-RUNX1T1 in primary HSPCs or cell lines resulted in down regulation of a number of genes involved in BER, including *OGG1* which encodes the primary enzyme responsible for excising oxidized bases (Alcalay et al., 2003; Krejci et al., 2008; Liddiard et al., 2010; Forster et al., 2016). RUNX1-RUNX1T1 was shown to occupy the *OGG1* gene and presumably directly regulates *OGG1* expression (Krejci et al., 2008; Forster et al., 2016). Functional defects in BER were observed in RUNX1-RUNX1T1 expressing cells, such as slow repair of DNA damage by alkylating agents (Alcalay et al., 2003) and accumulation of somatic mutations more rapidly over time (Forster et al., 2016).

Inefficient DNA repair by the HR pathway has also been documented in RUNX1-RUNX1T1 expressing cells. RUNX1-RUNX1T1 expressing cells are very sensitive to poly (ADP-ribose) polymerase (PARP) inhibitors (Esposito et al., 2015), which have potent activity specifically on cells with defective HR repair (Helleday, 2011). Treatment with PARP inhibitors suppressed the colony forming ability of RUNX1-RUNX1T1 expressing cells, consistent with a defect in HR repair (Esposito et al., 2015). A very early event in the repair of DNA double-strand breaks and a surrogate marker for breaks is phosphorylation of the histone variant H2AX (the phosphorylated form is γ-H2AX) by the ATM serine-threonine kinase (Mah et al., 2010). DNA double-strand breaks trigger a cascade of events that recruit DNA repair proteins, including the recombinase RAD51 through BRCA1 and BRCA2. RUNX1-RUNX1T1 expressing cells cultured *in vitro* gradually acquired more γ-H2AX foci than normal cells over time, indicating that they more rapidly accumulated unrepaired double-strand breaks (Krejci et al., 2008; Wichmann et al., 2015). Following acute induction of DNA damage, the γ-H2AX foci that formed disappeared more slowly, indicative of slower resolution or repair of γ-H2AX+ double strand breaks (Esposito et al., 2015). The slow repair of DNA correlated with decreased recruitment of the recombinase RAD51 to the DNA damage sites (Esposito et al., 2015). This deficiency in HR in RUNX1-RUNX1T1 expressing cells was associated with lower expression of genes responsible for HR, including *BRCA1, BRCA2, ATM*, and *RAD51* (Esposito et al., 2015). Whether RUNX1-RUNX1T1 regulates these DNA repair genes directly or indirectly is unclear.

Defects in DNA repair have also been observed with the types of *RUNX1* mutations found in FPD/AML. Retroviral transduction of a dominant negative truncated RUNX1 protein lacking its transactivation domain into HSPCs resulted in a greater proportion of cells with evidence of DNA damage, as detected by the presence of an increased number of cells containing γ-H2AX^+^ foci and slower decay of γ-H2AX^+^ foci following DNA damage induction (Satoh et al., 2012). The defect in DNA repair was accompanied by the downregulation of several DNA repair genes, including *RAD51* and the growth arrest and DNA damage gene *GADD45*. The RUNX proteins may also directly regulate the FA/BRCA pathway (Krejci et al., 2008; Wang et al., 2014). The FA/BRCA pathway is essential for repairing interstrand crosslinks in DNA. Inherited mutations in multiple FA/BRCA pathway genes cause bone marrow failure and predispose to myeloid leukemia and other cancers (Cheung and Taniguchi, 2017; Table 1). The FA/BRCA pathway is strongly activated by the crosslinking agent mitomycin C. When RUNX1 and its homolog RUNX3 were together deleted or knocked down, the sensitivity of cells to mitomycin C increased, indicating inefficient repair of the damage (Wang et al., 2014). RUNX1 was shown to co-immunoprecipitate with FANCD2, a protein in the FA/BRCA pathway that localizes to sites on interstrand crosslinks (Wang et al., 2014). Recruitment of FANCD2 to DNA decreased upon combined knockdown of RUNX1 and RUNX3, and repair was depressed (Wang et al., 2014).

In addition to direct effects on DNA repair pathways, *RUNX1* mutations may promote the survival of HSCs with somatic mutations. For example, lower p53 levels have been documented in RUNX1 deficient HSPCs (Cai et al., 2015). The p53 transcription factor protects cells from DNA damage by arresting the cell cycle to allow DNA repair to take place and by inducing apoptosis and senescence of unrepaired cells. The reduction in p53 protein levels in RUNX1 deficient HSCs was not accompanied by reduced *Tp53* mRNA levels; therefore, the mechanism of the reduction is post-transcriptional (Cai et al., 2015). Other studies showed that RUNX1 forms a complex with p300-p53 to acetylate p53 and thereby activate p53 target genes (Wu et al., 2013). Hence loss of RUNX1 could affect both p53 levels and activity. RUNX1 deficient HSCs contained a decreased percentage of apoptotic cells, both in the absence and presence of DNA damaging agents (Motoda et al., 2007; Cai et al., 2011, 2015). Thus, inefficient elimination of cells that have acquired DNA damage through impaired p53 activity (Cai et al., 2015) could contribute to the accumulation of HSCs with unrepaired DNA damage and acquired secondary mutations in FPD/AML. Other survival pathways and apoptotic pathways are also likely to be dysregulated in RUNX1 deficient HSCs, as increasing p53 levels by inhibiting its interaction with MDM2 using the MDM2:p53 inhibitor nutlin-3 did not completely correct the low apoptotic phenotype (Cai et al., 2015). All of the above-mentioned studies were performed with cells that have more severe perturbations in functional RUNX1 than would be expected with the mono-allelic mutations in FPD/AML, and the DNA repair and apoptotic defects are likely to be more profound in experimental models. Nevertheless, the median age for leukemia development in FPD/AML individuals is 33 years (Owen C. J. et al., 2008). Thus, even minor defects in DNA repair could contribute to the increased accumulation of mutations over a period of several decades.

## Forces that may drive clonal selection of HSCs with secondary mutations in FPD/AML

For an HSC with a somatic mutation to clonally expand, there must be selective pressures favoring that HSC that provide it with a competitive advantage. The nature of selective pressures can change with age; for example, the selective pressure that drives the expansion of somatically mutated cells is thought to increase with age and contribute to the age-associated increased incidence of cancer (Rozhok and DeGregori, 2016). An aged inflammatory niche, for instance, was a critical determinant in selecting for HSPCs with oncogenic driver mutations in mice (Henry et al., 2015). The ability of HSPCs transduced with oncogenes (*NRAS*^*V12*^*, BCR-ABL*, and *MYC*) to promote leukemogenesis was greatly enhanced by transplantation into old recipient mice, whereas when transplanted into young hosts, leukemogenesis was largely suppressed (Henry et al., 2015). The expansion of the transduced HSPCs in the aged bone marrow niche could be prevented by transgene expression of the anti-inflammatory mediators α-1-antitrypsin (AAT), or interleukin-37 (IL-37) (Henry et al., 2015). These findings suggest that not only do mutations need to be acquired in HSCs but also that the leukemic clone must be in the inflammatory environment of an aged bone marrow for the mutations to provide a fitness advantage that can be selected for. Consistent with this, recent studies showed that inflammatory cytokines can promote the selective *in vitro* growth of human AML cells. In a large functional screen of primary human AML samples, several inflammatory cytokines, including IL-1, promoted the growth of AML progenitors while suppressing the growth of normal HSPCs (Carey et al., 2017). Strikingly, despite genetic and clinical heterogeneity, the growth of 40 out of 60 primary AML samples was stimulated by IL-1, suggesting that selective growth driven by inflammatory cytokines is a common feature of many AML cells (Carey et al., 2017). Differentiated myeloid cells were identified as the primary source of IL-1, highlighting the critical role that an inflammatory niche may play in leukemogenesis (Carey et al., 2017).

Increased production of inflammatory cytokines by myeloid cells has been observed in individuals with CHIP and in mouse models of the mutations found in CHIP. Evidence of inflammation and increased serum levels of IL-8 were identified in CHIP associated with mutations in *TET2* (Jaiswal et al., 2017). Myeloid cells in mice were shown to be a source of inflammatory cytokines, as a myeloid cell specific disruption of *Tet2* with Lyz2-Cre elevated mRNAs encoding multiple chemokines and inflammatory cytokines; furthermore, endotoxin stimulation of *Tet2* deficient macrophages resulted in more robust secretion of IL-1β and IL-6 (Cull et al., 2017; Fuster et al., 2017; Jaiswal et al., 2017). Hence, mutations in HSCs may increase the production of inflammatory cytokines by downstream myeloid lineage cells, which could potentially feedback on and selectively promote the growth of the mutated HSCs.

Clinical evidence hints that elevated inflammation may contribute to disease severity in FPD/AML individuals. In one FPD/AML pedigree, affected family members were reported to have eczema, the severity of which correlated directly with the severity of thrombocytopenia (Sorrell et al., 2012). Strikingly, eczema was most severe in those family members who went on to develop frank leukemia (Sorrell et al., 2012). In another study, hypersensitivity to G-CSF was observed in peripheral blood mononuclear cells from an FPD/AML patient (Chin et al., 2016). This correlates with findings in mouse models, where increased sensitivity of HSPCs to G-CSF upon mono- or biallelic deletion of *Runx1* was proposed to result from an increase in STAT3 signaling (Chin et al., 2016; Lam et al., 2016).

Further suggestive evidence that mutations in RUNX1 may elevate inflammation can be seen in studies of its homolog RUNX3, which binds the same DNA sequence as RUNX1 and is a well-documented negative regulator of inflammation. As is well reviewed elsewhere, genome wide association studies (GWAS) linked *RUNX3* with diseases of inflammation in humans including ulcerative colitis, celiac disease, ankylosing spondylitis, psoriasis, and asthma (Lotem et al., 2015). *Runx3* knockout mice exhibit spontaneous colitis and lung inflammation, in part due to dysregulated TGF-β signaling (Brenner et al., 2004; Fainaru et al., 2004). RUNX1 also negatively regulates inflammatory signaling in the lung (Tang et al., 2017). Deletion of *Runx1* in lung epithelium resulted in constitutive activation of NF-κB pro-inflammatory signaling and increased susceptibility to LPS-induced acute lung injury *in vivo* (Tang et al., 2017). Given these findings in lung epithelium, germ line mutations in *RUNX1* could have more pleiotropic effects in patients with FPD/AML than previously appreciated.

The mechanisms by which RUNX1 regulates inflammation are unclear. RUNX1 is reported to have opposing effects on NF-κB signaling output due to interactions with different members of the NF-κB signaling pathway. Nakagawa *et al*. showed that RUNX1 interacted with the IκB kinase (IKK) complex in the cytoplasm, and deletion of *Runx1* activated NF-κB signaling (Nakagawa et al., 2011). The ability of RUNX1 to inhibit IKK activity was lost in three RUNX1 mutants identified in MDS patients (Nakagawa et al., 2011). However, a different group reported a contrasting activity of RUNX1 in regulating NF-κB, in that knock down of RUNX1 in myeloid cell lines and activated primary mouse peritoneal macrophages attenuated NF-κB signaling (Luo et al., 2016). The latter study also showed that RUNX1 binds to p50 (a NF-κB family member) in the nucleus and may lead to enhanced NF-κB signaling via recruitment of RNA polymerase II at some p50-occupied sites (Luo et al., 2016). Further, use of a RUNX1 inhibitor provided some protection against LPS-induced septic shock in an *in vivo* murine model (Luo et al., 2016). Therefore, RUNX1 appears to interface with the NF-κB pathway at multiple points and to be capable of both interfering with and augmenting NF-κB signaling.

RUNX proteins can also modulate inflammatory signaling through their regulation of T cell development and function (Djuretic et al., 2009; Wong et al., 2011). RUNX1 and RUNX3 influence the development and function of T_H_1, T_H_2, T_REG_, and T_H_17 cells, which regulate autoimmunity, asthma, allergic responses, and tumor immunity (Djuretic et al., 2007, 2009; Naoe et al., 2007; Ono et al., 2007; Zhang et al., 2008; Bruno et al., 2009; Wong et al., 2011). T cell specific deletion of *Runx1* caused spontaneous hyperactivation of CD4+ T cells resulting in severe lung inflammation that evolved into a lethal systemic inflammatory disease (Wong et al., 2012).

Putting all this together, we speculate that systemic inflammation may be elevated in FPD/AML and that an inflammatory bone marrow microenvironment may provide a positive selective pressure for HSCs that have acquired secondary mutations.

## Effects of *RUNX1* mutations on HSCs

The mutations in epigenetic regulator genes that initiate AML and predominate in CHIP have the capability of selectively expanding HSCs. This phenomenon of clonal HSC expansion has been reproduced in several mouse models. Deletion of *Dnmt3a* in mice skewed the balance of self-renewal versus differentiation of HSCs, causing an accumulation of HSCs in the bone marrow, inefficient output of HSCs to differentiated hematopoietic progeny in the periphery, and a repopulation advantage of *Dnmt3a* deficient HSCs over normal HSCs in transplant experiments (Challen et al., 2012; Shlush et al., 2014). Mutations in *Tet2* similarly conferred a competitive advantage to murine HSCs, allowing them to outcompete normal HSCs in transplantation experiments (Ko et al., 2011; Moran-Crusio et al., 2011; Quivoron et al., 2011).

RUNX1 mutant HSCs do not appear to enjoy the same competitive advantage over normal HSCs as do HSCs with *Dnmt3a* or *Tet2* mutations. *RUNX1* mutations may require the context of additional mutations in order to provide a fitness advantage for the leukemic clone. Initial reports showed that deletion of *Runx1* in hematopoietic cells caused an expansion of HSPCs (lineage negative Sca1^+^ Kit^+^ cells) and committed myeloid progenitors in the bone marrow (Ichikawa et al., 2004; Growney et al., 2005). However, the majority of studies in which careful analyses were carried out on functional long-term repopulating HSCs (LT-HSCs) reported modest, 2-4-fold decreases in their frequency upon *Runx1* mutation (Sun and Downing, 2004; Jacob et al., 2009; Cai et al., 2011). For example, a pan-hematopoietic homozygous deletion of *Runx1* resulted in a 3-4-fold decrease in functional LT-HSCs (measured in limiting dilution transplantation experiments) in the bone marrow of both young and aged mice (Jacob et al., 2009; Cai et al., 2011). Similarly, monoallelic germ line mutations in *Runx1* reduced the frequency of functional LT-HSCs in the bone marrow by about 50% (Sun and Downing, 2004). *Runx1* deficient (*Runx1*^Δ/Δ^), *Runx1*^+/−^, and wild type HSCs performed equivalently well in serial transplantation experiments, in that by the fourth round of transplantation, HSCs of all three genotypes were essentially exhausted, and hence had similar self-renewal potential (Sun and Downing, 2004; Cai et al., 2011). By comparison, *Dnmt3a* deficient HSCs expanded with each subsequent round of transplantation (Challen et al., 2012). Therefore, the inability of *RUNX1* mutations to induce clonal HSC expansion like that seen with *DNMT3A* and *TET2* mutations may account for the rarity of *RUNX1* mutations as initiating events in AML and in CHIP.

An interesting and potentially clinically consequential feature of RUNX1 mutant HSPCs is that they exhibit a greater selective advantage over normal HSPCs following exposure to radiation. Irradiation of a mixture of *Runx1* deficient (*Runx1*^Δ/Δ^) and normal bone marrow cells followed by transplantation into mice resulted in a greater selective expansion of *Runx1* deficient HSPCs (lineage negative Sca1^+^ Kit^+^ cells) and myeloid lineage cells in the bone marrow compared to irradiated wild type or non-irradiated *Runx1* deficient cells (Cai et al., 2015). This was likely due to the increased resistance of *Runx1* deficient HSPCs to endogenous and genotoxic stress (Cai et al., 2015). A very similar phenomenon of radiation-dependent HSC expansion was also observed in HSPCs with a *Tp53* mutation, consistent with this interpretation (Marusyk et al., 2010). *Runx1* deficient HSPCs were less apoptotic, grew more slowly than normal HSCs, had a lower rate of protein translation, and had markedly decreased ribosome biogenesis. Consistent with decreased ribosome biogenesis, *Runx1* deficient HSPCs were smaller than normal HSPCs, and preliminary data suggested that this small cell phenotype was shared with HSPCs from FPD/AML individuals (Cai et al., 2015). This stress resistant, slow growth, low apoptotic phenotype could presumably confer resistance to treatment and may explain why *RUNX1* mutations confer a relatively poor prognosis in AML. It may also explain the observation that *RUNX1* mutations occurred at a higher frequency in MDS patients previously exposed to low-level radiation as compared to all MDS patients (Harada et al., 2003; Zharlyganova et al., 2008).

## Conclusions

**Figure 1:**
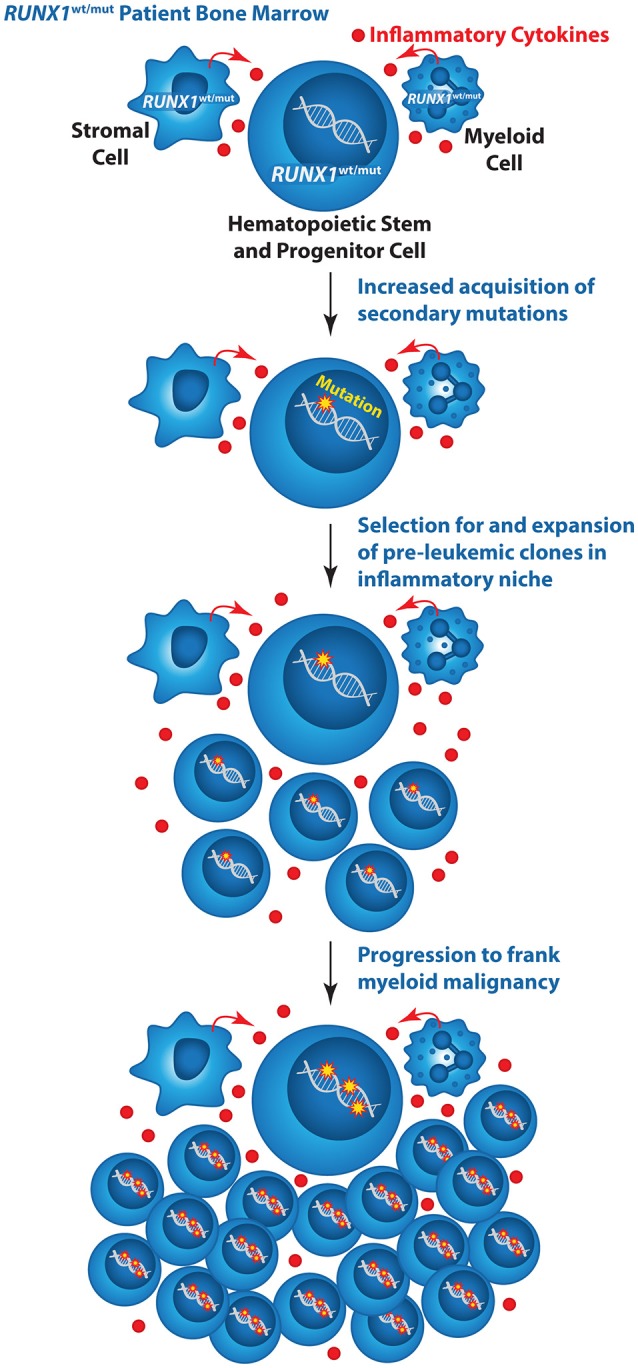
Model illustrating the mechanism by which inherited mutations in *RUNX1* could elevate the risk of AML in FPD/AML individuals. Wildtype (wt), mutant (mut).

It was originally thought that inherited *RUNX1* mutations cause leukemia predisposition because every HSC in an FPD/AML individual is already one mutation along the path to AML. However, it is likely that inherited *RUNX1* mutations play a more active role in promoting leukemia progression. The dysregulation of DNA repair and decreased apoptosis of RUNX1 mutant HSCs along with an increased inflammatory microenvironment may contribute to the markedly increased incidence of MDS, AML, and T-ALL in FPD/AML individuals. We envision a model whereby *RUNX1* mutations increase the rate at which secondary mutations are acquired; moreover, increased inflammatory signals delivered by RUNX1 mutant myeloid lineage cells or other cells in the RUNX1 mutant bone marrow microenvironment provide a selective pressure that confers a competitive advantage to FPD/AML HSCs that have acquired secondary mutations (Figure 1). On the other hand, in a normal individual a single HSC with a *RUNX1* mutation does not have a competitive advantage over normal HSCs and hence does not expand and initiate leukemia.

The initiation and evolution of leukemia in FPD/AML individuals can be carefully followed by next generation sequencing. Individuals diagnosed with FPD/AML could be monitored on a regular basis for evidence of clonal HSC expansion driven by acquired secondary mutations and treated before they progress to frank AML. However, we still do not know which secondary mutations are the most deleterious and whether they are the same as or different than the co-occurring mutations in sporadic AML. For example, *DNMT3A* mutations have been reported in two asymptomatic FPD/AML individuals (Churpek et al., 2015; Antony-Debré et al., 2016). Does the presence of a *DNMT3A* mutation, which in a normal individual only modestly increases the risk of leukemia, confer a much greater risk in the context of an inherited *RUNX1* mutation? More data and longitudinal studies correlating the stepwise accumulation of mutations in individuals with FPD/AML with clinical outcome are needed to predict which mutations most likely portend a rapid progression to MDS or AML. Mechanistic studies in animal and cell models will also be necessary to understand what is driving leukemia progression in FPD/AML and to identify strategies that can avert, delay, or reverse this progression.

## Author contributions

All authors listed have made a substantial, direct and intellectual contribution to the work, and approved it for publication.

### Conflict of interest statement

The authors declare that the research was conducted in the absence of any commercial or financial relationships that could be construed as a potential conflict of interest.
